# Direct observation of dual-step twinning nucleation in hexagonal close-packed crystals

**DOI:** 10.1038/s41467-020-16351-0

**Published:** 2020-05-18

**Authors:** Yang He, Bin Li, Chongmin Wang, Scott X. Mao

**Affiliations:** 1 Department of Mechanical Engineering and Materials Science, University of Pittsburgh, Pittsburgh, PA 15261 USA; 2 Department of Chemical and Materials Engineering, University of Nevada, Reno, NV 89557 USA; 3 Environmental Molecular Science Laboratory, Pacific Northwest National Laboratory, Richland, WA 99352 USA

**Keywords:** Structural properties, Metals and alloys

## Abstract

Design and processing of advanced lightweight structural alloys based on magnesium and titanium rely critically on a control over twinning that remains elusive to date and is dependent on an explicit understanding on the twinning nucleation mechanism in hexagonal close-packed (HCP) crystals. Here, by using in-situ high resolution transmission electron microscopy, we directly show a dual-step twinning nucleation mechanism in HCP rhenium nanocrystals. We find that nucleation of the predominant {1 0 −1 2} twinning is initiated by disconnections on the Prismatic│Basal interfaces which establish the lattice correspondence of the twin with a minor deviation from the ideal orientation. Subsequently, the minor deviation is corrected by the formation of coherent twin boundaries through rearrangement of the disconnections on the Prismatic│Basal interface; thereafter, the coherent twin boundaries propagate by twinning dislocations. The findings provide high-resolution direct evidence of the twinning nucleation mechanism in HCP crystals.

## Abstract (web summary)

Aspects of twinning in hexagonal-close-packed crystals remain elusive. Here, the authors directly image twinning in rhenium nanocrystals and show the process is mediated by disconnections on Prismatic│Basal interfaces as the twin initially deviates from its ideal orientation before it is corrected.

## Introduction

Twinning, on par with dislocation, is an essential carrier of crystal deformation^1,2^. Particularly, in hexagonal close-packed crystals (e.g., magnesium, titanium, and rhenium), it is required to mediate deformation along the <***c*** > axis of the crystal structure^3–5^. By creating coherent twin boundaries (CTB) within a crystal, twinning can significantly affect the physical properties of the crystal. For instances, twin architectures can confine dislocation activities and effectively increase the strength of metals without sacrificing their ductility^6–9^. As such, the atomic mechanism of twinning nucleation—the key to control twinning—has drawn extensive research interest^4,10–16^. Unfortunately, owing to a lack of direct experimental evidence, current knowledge on twinning nucleation in HCP crystals remains at the level of debatable theories.

Given that the reliability of simulations and topological analysis critically depends on a correct description of interatomic potentials^17,18^, and that ex situ static characterization may be misleading owing to the high tendency of detwinning upon unloading^19,20^, it is generally believed that in situ atomic scale investigations are indispensable for unraveling the mystery of twinning nucleation. As more than one atom exists in the motif of the HCP crystal, shear alone cannot move all atoms to their correct positions in the twin; additional atomic adjustments (called shuffles) are always required in the twinning process^17^, which not only significantly affects the dynamic process of twinning^13,18,21,22^ but also poses tremendous challenges on direct atomic interrogation.

Here, by using advanced crystal manipulation techniques and in situ high-resolution transmission electron microscopy (HRTEM), twinning nucleation processes in HCP rhenium nanocrystals are directly captured at atomic resolution. It is revealed that the {1 0 −1 2} <1 0 −1 −1> twinning nucleates through a dual-step mechanism lead by transformations of parent prismatic (P) planes into the twin basal (B) planes; this process establishes the lattice correspondences of the twin plus a minor rotational deviation from the ideal parent–twin mutual-orientation; subsequently, ideal twin forms by the rearrangement of interfacial defects on the P│B interfaces and ensuing formation of CTB. The findings provide direct evidences to the twinning nucleation mechanism in HCP crystals.

## Results

### Prismatic-to-basal transformation

**Fig. 1:**
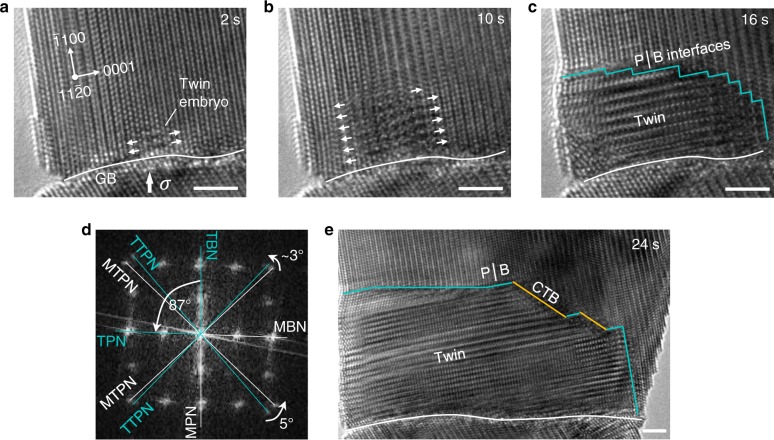
Direct observation of a twinning nucleation process. **a**–**c** Sequential HRTEM images showing twinning nucleation in a HCP rhenium nanocrystal under < 1 −1 0 0 > -oriented compression. Arrows indicate the expansion of basal layers in the twin embryo. Turquoise lines indicate the Prismatic│Basal (P│B) interfaces between the twin embryo and matrix. White lines indicate the grain boundary (GB). Block arrow in **a** indicates the loading direction. **d** Fast Fourier transformation of the twin embryo in **c**. TBN, MPN, TPN, MBN, TTPN, and MTPN mean the plane normal directions of twin basal, matrix prismatic, twin prismatic, matrix basal, twin twinning plane and matrix twinning plane, respectively. **e** Morphology of the twin as it grew. Yellow lines indicate the coherent twin boundaries (CTB). Scale bars in **a**–**c**, **e**, 2 nm.

Fig. 1a (and Supplementary Movie 1) shows a {1 0 −1 2} twinning nucleation process from the grain boundary of a rhenium (Re) bi-crystal under compression. Incipient plastic deformation started with the nucleation of a {1 0 −1 2} twin embryo at a local region of the grain boundary (where stress concentration might be present). The twin embryo was ~1 nm in dimension upon nucleation, i.e., containing only a few unit-cells, and grew by formation and lateral expansion of new basal layers on the twin/matrix interface (Fig. 1a–c). As it grew through the sample thickness direction, atomic-resolution image of the twin embryo was captured (see Fig. 1c), demonstrating that the laterally expanding layers were indeed basal planes. During this process, the {1 0 −1 2} twinning planes in the parent and twin crystals were not parallel (Fig. 1d), implying that the observed process in Fig. 1a–c was not dominated by twinning dislocations^1,23^. As the twin embryo grew in subsequent compression, CTB on the {1 0 −1 2} plane were formed (Fig. 1e), demonstrating that the prior transformation was indeed an incipient stage of the twinning nucleation. Same mechanism of twinning nucleation was also identified frequently on the side surfaces of different samples (see Supplementary Fig. 1).

**Fig. 2:**
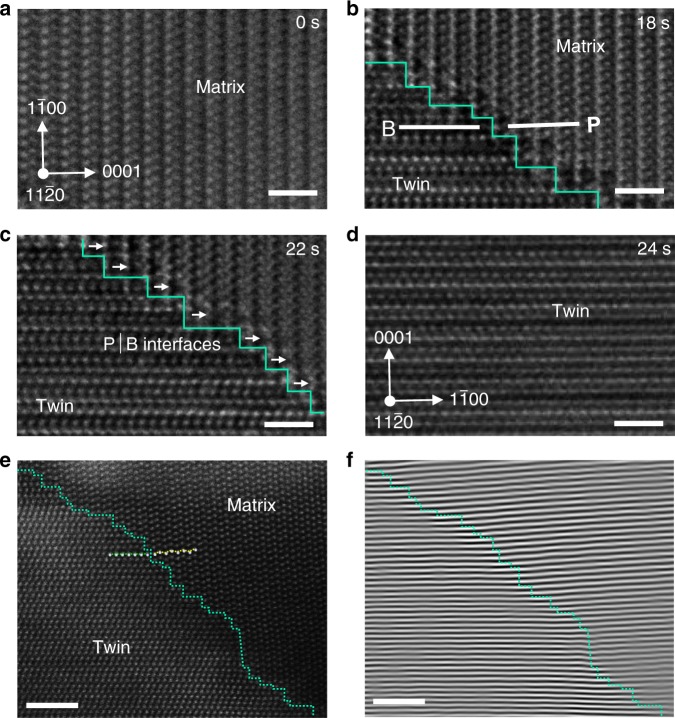
Twin embryo growth mediated by disconnections on the P│B interfaces. **a**–**d** Sequential HRTEM images showing the process. The zig-zag turquoise lines indicate the P│B interfaces, wherein the step features are disconnections on the P│B interface. Arrows indicate the moving direction of the disconnections. **e** Atomic-resolution HAADF-STEM image of the P│B-type twin boundaries and **f** corresponding inverse fast Fourier transformation showing one-to-one correspondence between twin basal planes and matrix prismatic planes. Insets in **e**) show the atomic models of the two types of plane. The zig-zag turquoise lines in **e**, **f** indicate the P│B interfaces. Scale bars in **a**–**d**, 1 nm; scale bars in **e**–**f**, 2 nm.

Zoom-in view of another {1 0 −1 2} twinning nucleation process demonstrates that expansion of the twin basal layers is actually mediated by disconnections on the P│B interfaces^24^ (Fig. 2a–d). Most of these disconnections showed a step height of two atomic layers (Fig. 2b–c); during their migration, the parent prismatic planes transformed into twin basal planes (P → B transformation) following a one-to-one planar correspondence (as evidenced by the atomic-resolution images in Fig. 2e–f).

### The deviation from an ideal {1 0 −1 2} twin

**Fig. 3:**
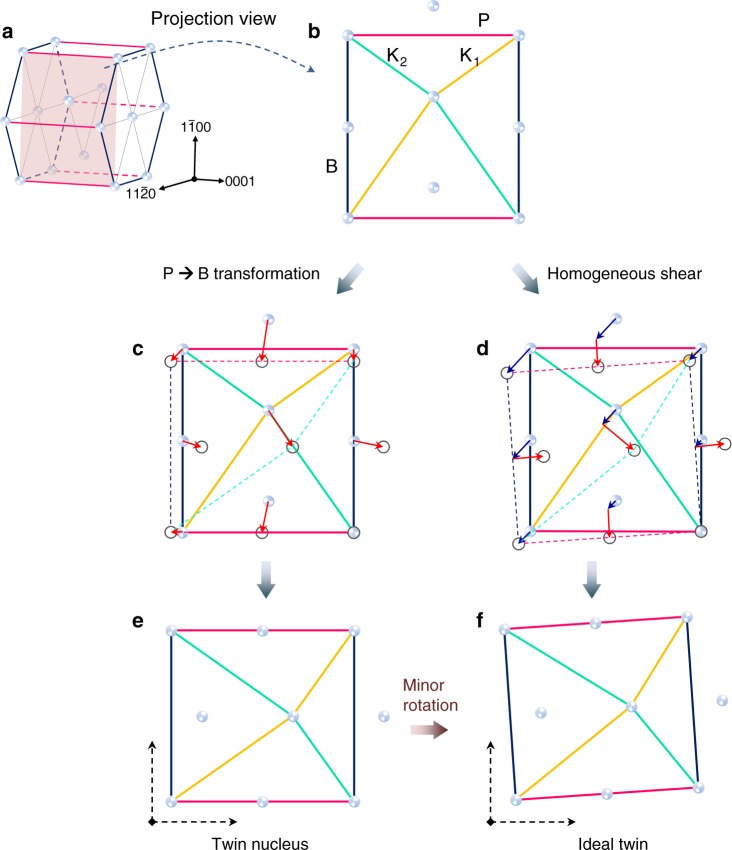
Schematic illustration of the dual-step twinning nucleation mechanism. **a** Unit-cell of a HCP structure. Solid balls represent individual atoms. **b** <1 1 −2 0 > -projection view of the unit-cell. The prismatic plane (P), basal plane (B), twinning plane (K_1_), and conjugate twinning plane (K_2_) are colored magenta, blue, yellow, and turquoise, respectively, for easy tracking of their corresponding planes after the transformations. Arrows in **c**, **d** indicate the atomic movements required to accomplish the transformations of the unit-cell; in particular, blue arrows in **d** indicate the portion that delivers the homogeneous shear. **e** Twin embryo structure generated by the P → B transformation, which shows a minor rotational deviation compared with the ideal twin **f**. Angle of the minor rotation can be described by tan^−1^(***s***/2), where ***s*** is the magnitude of twinning shear (see Supplementary Fig. 2 for the derivation).

Though the P → B transformation does not establish ideal parent–twin mutual-orientation (Fig. 1d), it establishes exactly the same lattice correspondence as that of an ideal {1 0 −1 2} twin^25^ (as schematically depicted by unit-cell level analysis in Fig. 3). To track the lattice correspondences, the prismatic plane, basal plane, twinning plane, and conjugate twinning plane are color-coded (Fig. 3a–b). In the observed mechanism, the parent P plane transforms into twin B plane, and the parent B plane transforms into twin P plane (Fig. 3c). As such, the twinning plane is rotated by ~4^o^ during the transformation. By contrast, position of the twinning plane is invariant in the ideal {1 0 −1 2} twinning (Fig. 3d). Clearly, the lattice correspondences generated by the two mechanisms are exactly the same (Fig. 3e–f), except for a minor rotation depending on the magnitude of twinning shear (Supplementary Fig. 2). In other words, the P → B transformation completes the twinning process in the sense that it moves all atoms in parent structure to their correct positions in the twin.

### Correction of the deviation

**Fig. 4:**
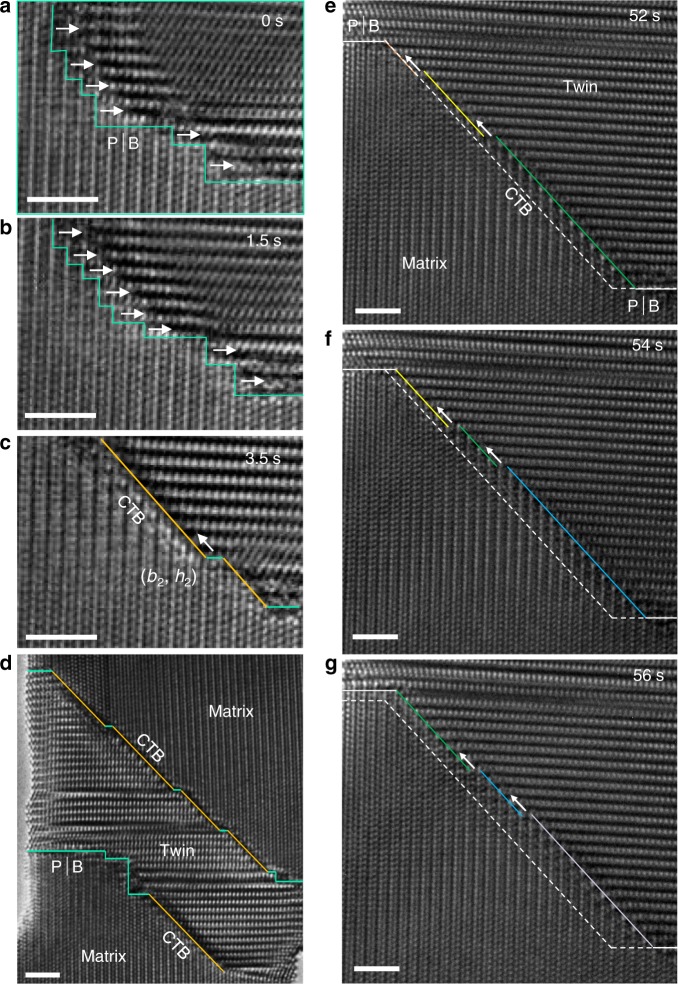
Twin boundary transformation from P│B interfaces to CTB during detwinning. **a**–**c** Sequential HRTEM snapshots of the process. The dominant defects in the twinning transit from disconnections on P│B interfaces to twinning dislocations (***b***_2_, *h*_2_) on CTB. Zig-zag turquoise lines indicate the P│B interfaces. White arrows in **a**, **b** indicate the moving directions of the disconnections on the P│B interface. **d** Overview of the twin upon partial detwinning, showing serrated twin boundaries. **e**–**g** Sequential HRTEM snapshots showing CTB propagation by twinning dislocations. White dash line is a reference line to show the migration of the CTBs (colored lines). Arrows in **c**, **e**–**g** indicate the migration direction of the twinning dislocations. Scale bars in **a**–**g**, 2 nm.

Interestingly, the minor rotational deviation from the ideal twin–parent mutual-orientation can be corrected by subsequent transformations. As shown in Fig. 4a–c, the disconnections on the P│B interfaces rearranged to align on the twinning plane upon unloading, forming CTB. Same transformation of twin boundaries can also happen during the twin embryo growth (Fig. 1e). This is essentially a twin boundary faceting process driven by twin boundary energy minimization^14,24,26,27^. More importantly, the orientation relation between the twin and matrix became ideal at very local regions thereafter (see Supplementary Fig. 3). Subsequent movement of the CTB was mediated by conventional (***b***_2_, *h*_2_) twinning dislocations (see Fig. 4d–g for that in a detwinning process and Supplementary Fig. 4 for that in a twinning process), manifesting a long-desired direct evidence to the classical mechanism of {1 0 −1 2} twinning^14,22,28^.

### The dual-step mechanism of twinning nucleation

In retrospect, above observations directly demonstrate a dual-step mechanism of the {1 0 −1 2} twinning nucleation: (I) establishment of the lattice correspondence through P → B transformation, and then (II) correction of the minor rotational deviation through twin boundaries transformation. Note that the formation process of the incipient unit-cell-scale twin embryo (Fig. 1a) is shrouded by the parent lattice along the TEM electron beam direction; based on the observation that it is a P → B transformation process, the unit-cell-scale embryo is likely formed by the pure-shuffle mechanism^11,14^.

It should be noted that the side surface of the nanocrystal can facilitate the P → B transformation by providing accommodation for the lateral deformation especially when the transformation propagates to the side surface. This is evidenced by the bulge on the left-side surface in Fig. 1c and the fact that P → B transformation played a major role in mediating the {1 0 −1 2} twinning nucleation and growth in our nanosized samples and CTB is only occasionally formed during the process. In the confined environment of bulk samples, accommodation mechanisms other than the side surface are needed to facilitate the P → B transformation, such as elastic and plastic deformation in front of the twin boundary^29^, deformation of the neighboring grains^30^, intersection region of the grain boundaries^31^ and formation of threading dislocations on the P│B interface^27,32^. Considering the fact that the incipient nucleation is a local event at atomic scale, one or more of these mechanisms should be able to accommodate the limited deformation, resulting from the initial P → B transformation as a leading step of twinning nucleation. This is supported by the fact that initial nucleus of the twin in our experiment is away from (and without obviously bulging) the side surface (Fig. 1a–b). Above all, the dual-step manner of the twinning nucleation should persist in the interior of the bulk samples. Though, the energy barrier for the same mechanism happening close to the free surface or in the bulk of the aggregate should be different. Compared with the free side surface, the above strain accommodation mechanisms in bulk samples likely requires a higher stress to be active; therefore, growth of the twin nucleus by continuous P → B transformation may be limited and the classical shear of twinning dislocations on the CTB may become prevalent in bulk samples.

In addition, our observation does not disprove other twinning nucleation models (e.g., reaction and dissociation of matrix dislocations^33–36^, surface/interface emission of twinning dislocations^10,30,37,38^); the nanoscale dimension of the crystals here prompts the starvation of matrix dislocations^39–41^, eliminating potential twinning nucleation through the pole-mechanism^33,34^, or dislocation dissociation^35,36^. It may also inhibit the nucleation of twinning dislocations from the side surface^41^. Moreover, the bi-nanocrystal sample geometry has much less grain boundaries than a bulk polycrystalline sample and may significantly reduce the availability of favorable nucleation sites for twinning dislocations^10,42^.

## Discussion

Though our finding is based on Re nanocrystals, it can be reasonably extended to other bulk HCP metals. First, the observations provide a direct understanding on the origin of the serrated twin boundaries in many bulk HCP metals including Co^21,43–46^, Ti^47,48^, Mg^32,49,50^, Zn^29,51,52^. Second, it is well known that *c/a* ratio has critical influence on the deformation of HCP metals^5^; as the *c/a* ratio of Re (1.615) is very close to those of Mg (1.624) and Ti (1.588), it is reasonable to expect the observed twinning nucleation mechanism in these metals (see Supplementary Fig. 2 for further discussion). Third, the dual-step twinning nucleation mechanism offers another fundamental understanding to the fact that the active twinning variant in HCP metals is not always associated with the largest resolved shear stress on the twinning plane^18,42,53^. As the onset of P → B transformation depends on the normal stresses on the prismatic/basal planes which, in most cases, translate to both a resolved shear stress and a normal stress on the twinning plane, twinning nucleation by the dual-step mechanism can also be critically affected by the normal stress on the twinning plane. Finally, a major advantage of nanoscale samples over bulk sample is that high-stress states can be attained^54^; localized stress concentrations within bulk samples may provide equivalent stress condition for the activation of the dual-step mechanism.

**Fig. 5:**
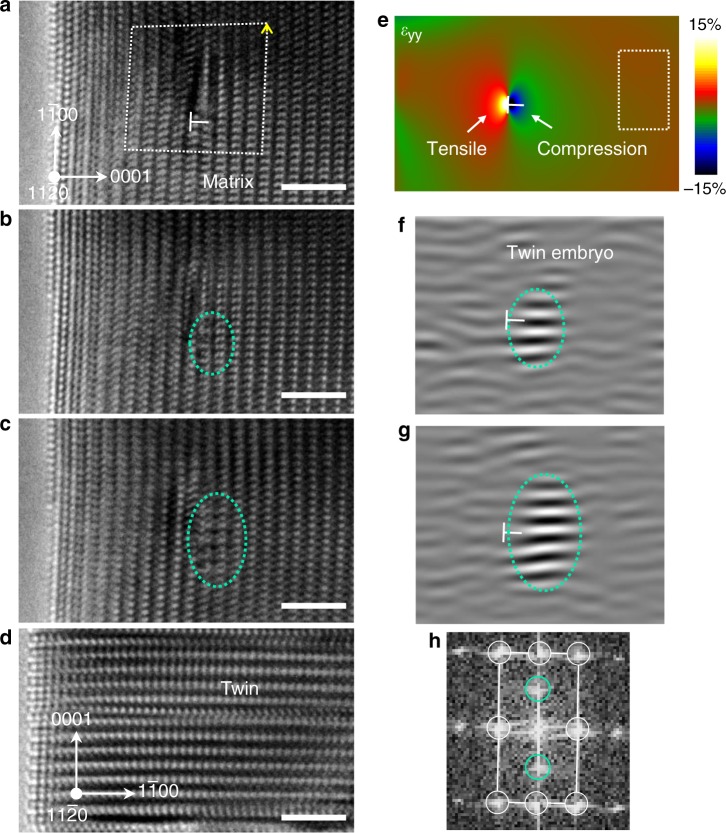
Twinning nucleation assisted by the strain field of a matrix dislocation. **a**–**d** Sequential HRTEM images showing the nucleation of a twin embryo near the dislocation core (indicated by the “├” symbol). The crystal was under <1 −1 0 0 > -oriented compression and viewed along the <1 1 −2 0> direction. White dashed line in **a** is the Burgers circuit which indicates an <***a*** > component in the Burgers vector (indicated by the yellow arrow) of the dislocation. Detailed analysis of the Burgers vector is shown in Supplementary Fig. 5. **e** Mapping of the strain normal to the parent prismatic plane (*ε*_*yy*_) generated by the dislocation core, by geometrical phase analysis. Boxed region is the reference region for the geometrical phase analysis (see ref. ^56^ for more details). **f**–**g** Inverse fast Fourier transformations of the TEM snapshots in **b**, **c**, respectively, using the (0 0 0 1) reflections of the twin (indicated by turquoise-circles in **h**). Turquoise dotted circles in **b**, **c**, **f**, **g** mark the twin embryo. White circles and lines in **h** indicate the reciprocal lattice of the matrix. Scale bars in **a**–**d**, 2 nm.

Moreover, our findings provide a solid fundamental basis for the twinning-based design and processing of advanced HCP alloys. Since the {1 0 −1 2} twinning starts at low stress-state and has crucial role in the early stage deformation of magnesium alloys^18,55^, it is critical to impede the easy nucleation or growth of {1 0 −1 2} twins for strengthening the magnesium alloys. Based on the findings here, an effective way to do this is through cyclic deform-unload-anneal processing. In the deform-unload process, {1 0 −1 2} twins nucleates and propagate; upon unloading, the twin boundaries retreat and relocate to the CTB (as demonstrated in Fig. 4 and Supplementary Fig. 3). In the subsequent anneal process, the alloying elements will gradually segregate on the CTB^6^ and pin these boundaries from further propagation. Then, the future deformation would require nucleation of new {1 0 −1 2} twins rather than propagation of existing {1 0 −1 2} twins. As such, the easy nucleation sites for {1 0 −1 2} twinning can be gradually depleted by cyclic deform-unload-anneal processing. Increasing the interfacial energy of P│B-type interfaces^27^ (e.g., by alloying) raises the formation energy of the twin embryo and hence makes it harder for the {1 0 −1 2} twinning nucleation through the observed mechanism. Moreover, hierarchical twin structures may be enabled by patterning desired defects for twinning nucleation. Per Fig. 3c, the P → B transformation correlates to a compressive strain on the prismatic plane, suggesting that normal strain fields from the defects may be used to control the twinning nucleation. Consistently, we captured a twinning nucleation event at the compressive strain field of a dislocation core (Fig. 5 and Supplementary Fig. 5). As revealed by quantitative geometrical phase analysis^56^ (Fig. 5e), the dislocation in Fig. 5a generated compressive strains on the prismatic planes to the right of the dislocation core; in the subsequent compression of the crystal, a {1 0 −1 2} twinning preferentially nucleated at the region where compressive strain of the dislocation core was the largest, demonstrating that the strain field of matrix dislocations could prompt the twinning nucleation. It should be noted that a high compressive stress (~5 GPa based on a rough estimation^57^) was attained at the moment of the twinning nucleation in Fig. 5b, which is not readily available in polycrystalline Re samples without significant stress concentrations^30^. Though, similar mechanism is expected to be present in nano-grains of HCP metals wherein high-stress-state can be attained.

Through direct atomic scale observation, we discovered that nucleation of the predominant {1 0 −1 2} twinning is completed in two steps: I. Establishing the lattice correspondence through an interfacial process that directly transforms the parent prismatic plane into a twin basal plane; II. Correction of residual misorientation through rearrangement of the interfacial defects and ensuing formation of CTBs. In addition, the role of matrix dislocations on the twinning nucleation and direct evidence to the operation of the classical twinning dislocations are also revealed. The mechanism is expected to be present in bulk HCP metals, providing a solid fundamental basis for the twinning-based design and processing of HCP alloys. Fundamentally, this mechanism suggests that nucleation of twinning (or martensitic transformation) is essentially a transformation process that conforms to and establishes the lattice correspondence, which can be accomplished by many interfacial processes other than the classical twinning dislocations on the twinning (or habit) plane.

## Methods

### In situ TEM experimental procedures

Rhenium is widely used in structure materials in jet engines^58^, with outstanding mechanical properties and excellent oxidation resistances at high temperatures. Moreover, Re is resistant to electron irradiation^59^, making it a suitable model material for in situ TEM investigations. Re rods with 99.999% purity (ESPI metals) were cut into nanosized tips and loaded onto both the fixed-end and the piezo-end of a Nanofactory holder. Then, the nanotips were manipulated to touch each other inside the high vacuum (~10^−8^ mbar) of a TEM and welded together by using an electric pulse. Then, the crystals were deformed by straining at a controlled rate of 10^−3^~10^−4^/s. FEI 80-300 Titan equipped with a imaging lens spherical aberration corrector was used throughout this work. A charge-coupled device was used to record the images and videos (at two frames per second).

## Supplementary information

Supplementary Information

Description of Additional Supplementary Files

Supplementary Movie 1

## Data Availability

All the data related to this manuscript have been included in the main text and supplementary information. All the raw data are stored in Environmental Molecular Science Laboratory at Pacific Northwest National Laboratory and is available upon reasonable request.

## References

[1] Christian JW, Mahajan S (1995). Deformation twinning. Prog. Mater. Sci..

[2] Zhu YT, Liao XZ, Wu XL (2012). Deformation twinning in nanocrystalline materials. Prog. Mater. Sci..

[3] Wu Z, Curtin WA (2015). The origins of high hardening and low ductility in magnesium. Nature.

[4] Britton TB, Dunne FPE, Wilkinson AJ (2015). On the mechanistic basis of deformation at the microscale in hexagonal close-packed metals. Proc. R. Soc. A.

[5] Yoo MH (1981). Slip, twinning, and fracture in hexagonal close-packed metals. Met Trans. A.

[6] Nie JF, Zhu YM, Liu JZ, Fang XY (2013). Periodic segregation of solute atoms in fully coherent twin boundaries. Science.

[7] Lu L, Chen X, Huang X, Lu K (2009). Revealing the maximum strength in nanotwinned copper. Science.

[8] Zhang Z (2017). Dislocation mechanisms and 3D twin architectures generate exceptional strength-ductility-toughness combination in CrCoNi medium-entropy alloy. Nat. Commun..

[9] Fu H (2017). Achieving high strength and ductility in magnesium alloys via densely hierarchical double contraction nanotwins. Nano Lett..

[10] Wang J, Hirth JP, Tomé CN (2019). Twinningnucleation mechanisms in hexagonal-close-packed crystals. Acta Mater.

[11] Wang J, Yadav SK, Hirth JP, Tomé CN, Beyerlein IJ (2013). Pure-shuffle nucleation of deformation twins in hexagonal-close-packed metals. Mater. Res. Lett..

[12] Serra A, Bacon DJ, Pond RC (2010). Comment on “atomic shuffling dominated mechanism for deformation twinning in magnesium”. Phys. Rev. Lett..

[13] Li B, Ma E (2009). Atomic shuffling dominated mechanism for deformation twinning in magnesium. Phys. Rev. Lett..

[14] Hirth JP, Wang J, Tomé CN (2016). Disconnections and other defects associated with twin interfaces. Prog. Mater. Sci..

[15] Ishii A, Li J, Ogata S (2016). Shuffling-controlled versus strain-controlled deformation twinning: the case for HCP Mg twin nucleation. Int J. Plasticity.

[16] Beyerlein IJ, Zhang X, Misra A (2014). Growth twins and deformation twins in metals. Annu Rev. Mater. Res..

[17] Bilby BA, Crocker AG (1965). The theory of the crystallography of deformation twinning. Proc. R. Soc. A.

[18] Barrett CD, El Kadiri H, Tschopp MA (2012). Breakdown of the Schmid law in homogeneous and heterogeneous nucleation events of slip and twinning in magnesium. J. Mech. Phys. Solids.

[19] Cáceres CH, Sumitomo T, Veidt M (2003). Pseudoelastic behaviour of cast magnesium AZ91 alloy under cyclic loading–unloading. Acta Mater..

[20] Proust G, Tomé CN, Jain A, Agnew SR (2009). Modeling the effect of twinning and detwinning during strain-path changes of magnesium alloy AZ31. Int J. Plasticity.

[21] Zhang XY, Li B, Tu J, Sun Q, Liu Q (2015). Non-classical twinning behavior in dynamically deformed cobalt. Mater. Res. Lett..

[22] Khater HA, Serra A, Pond RC (2013). Atomic shearing and shuffling accompanying the motion of twinning disconnections in zirconium. Philos. Mag..

[23] Li B, Zhang XY (2016). Twinning with zero twinning shear. Scr. Mater..

[24] Barrett CD, El Kadiri H (2014). The roles of grain boundary dislocations and disclinations in the nucleation of {10–12} twinning. Acta Mater..

[25] Niewczas M (2010). Lattice correspondence during twinning in hexagonal close-packed crystals. Acta Mater..

[26] Barrett CD, El Kadiri H (2014). Impact of deformation faceting on {10–12}, {10–11} and {10–13} embryonic twin nucleation in hexagonal close-packed metals. Acta Mater..

[27] Xu B, Capolungo L, Rodney D (2013). On the importance of prismatic/basal interfaces in the growth of twins in hexagonal close packed crystals. Scr. Mater..

[28] Serra A, Bacon DJ, Pond RC (1988). The crystallography and core structure of twinning dislocations in H.C.P. metals. Acta Met..

[29] Braisaz T, Ruterana P, Nouet G (1997). Twin tip defects related to the nucleation and growth mechanisms of the twin (1012) in zinc characterized by high-resolution electron microscopy. Philos. Mag. A.

[30] Kacher J, Minor AM (2014). Twin boundary interactions with grain boundaries investigated in pure rhenium. Acta Mater..

[31] Shekhar S, King AH (2008). Strain field and energies of grain boundary triple junctions. Acta Mater..

[32] Liu BY (2014). Twinning-like lattice reorientation without a crystallographic twinning plane. Nat. Commun..

[33] Yu Q (2010). Strong crystal size effect on deformation twinning. Nature.

[34] Thompson N, Millard DJ (1952). Twin formation in cadmium. Philos. Mag..

[35] Mendelson S (1970). Dislocation dissociations in hcp metals. J. Appl Phys..

[36] Ghazisaeidi M, Curtin WA (2013). Analysis of dissociation of <c> and <c+a> dislocations to nucleate (10-12) twins in Mg. Model. Simul. Mater. Sci. Eng..

[37] Yu Q, Qi L, Chen K, Mishra RK, Li J, Minor AM (2012). The nanostructured origin of deformation twinning. Nano Lett..

[38] Wang J, Zeng Z, Weinberger CR, Zhang Z, Zhu T, Mao SX (2015). In situ atomic-scale observation of twinning-dominated deformation in nanoscale body-centered cubic tungsten. Nat. Mater..

[39] Shan ZW, Mishra RK, Syed Asif SA, Warren OL, Minor AM (2008). Mechanical annealing and source-limited deformation in submicrometre-diameter Ni crystals. Nat. Mater..

[40] Uchic MD, Dimiduk DM, Florando JN, Nix WD (2004). Sample dimensions influence strength and crystal plasticity. Science.

[41] Zhu T, Ju L, Samanta A, Leach A, Gall K (2008). Temperature and strain-rate dependence of surface dislocation nucleation. Phys. Rev. Lett..

[42] Beyerlein IJ, Capolungo L, Marshall PE, McCabe RJ, Tomé CN (2010). Statistical analyses of deformation twinning in magnesium. Philos. Mag..

[43] Tu J, Zhang X, Wang J, Sun Q, Liu Q, Tomé CN (2013). Structural characterization of {10-12} twin boundaries in cobalt. Appl. Phys. Lett..

[44] Zhang XY, Tu J, Liu Q (2012). High-resolution electron microscopy study of the twin boundary and twinning dislocation analysis in deformed polycrystalline cobalt. Scr. Mater..

[45] Tu J, Zhang XY, Lou C, Liu Q (2013). HREM investigation of twin boundary and interface defects in deformed polycrystalline cobalt. Philos. Mag. Lett..

[46] Wang J, Liu L, Tomé CN, Mao SX, Gong SK (2013). Twinning and de-twinning via glide and climb of twinning dislocations along serrated coherent twin boundaries in hexagonal-close-packed metals. Mater. Res Lett..

[47] Sun Q, Zhang XY, Tu J, Ren Y, Qin H, Liu Q (2015). Characterization of basal-prismatic interface of twin in deformed titanium by high-resolution transmission electron microscopy. Philos. Mag. Lett..

[48] Braisaz T (1996). High-resolution electron microscopy study of the (1012) twin and defects analysis in deformed polycrystalline alpha titanium. Philos. Mag. Lett..

[49] Sun Q, Zhang XY, Ren Y, Tu J, Liu Q (2014). Interfacial structure of {10-12} twin tip in deformed magnesium alloy. Scr. Mater..

[50] Liu B-Y, Wan L, Wang J, Ma E, Shan Z-W (2015). Terrace-like morphology of the boundary created through basal-prismatic transformation in magnesium. Scr. Mater..

[51] Braisaz T, Ruterana P, Lebouvier B, Nouet G (1995). Atomic Structure Analysis of the (10-12) Twin in Zinc by HRTEM and Energetical Calculations. Phys. Stat. Sol..

[52] Braisaz T, Ruterana P, Nouet G, Pond RC (1997). Investigation of {1012} twins in Zn using high-resolution electron microscopy: Interfacial defects and interactions. Philos. Mag. A.

[53] Capolungo L, Marshall PE, McCabe RJ, Beyerlein IJ, Tomé CN (2009). Nucleation and growth of twins in Zr: a statistical study. Acta Mater..

[54] Liu BY (2019). Large plasticity in magnesium mediated by pyramidal dislocations. Science.

[55] Yu Q, Zhang J, Jiang Y (2011). Direct observation of twinning–detwinning–retwinning on magnesium single crystal subjected to strain-controlled cyclic tension–compression in [0001] direction. Philos. Mag. Lett..

[56] Hytch MJ, Snoeck E, Kilaas R (1998). Quantitative measurement of displacement and strain fields from HRTEM micrographs. Ultramicroscopy.

[57] Tromans D (2011). Elastic anisotropy of HCP metal crystals and polycrystals. IJRRAS.

[58] Carlen JC, Bryskin BD (1994). Rhenium - a unique rare metal. Mater. Manuf. Process..

[59] Egerton RF, Li P, Malac M (2004). Radiation damage in the TEM and SEM. Micron.

