# Supplementary material for: Direct observation of dual-step twinning nucleation in hexagonal close-packed crystals
Source: Nat Commun. 2020 May 18;11:2483. doi: 10.1038/s41467-020-16351-0 (PMC7235251; doi:10.1038/s41467-020-16351-0)
Supplement: Supplementary file 1 — Supplementary Information [file 41467_2020_16351_MOESM1_ESM.pdf]

Direct Observation of Dual-Step Twinning Nucleation in Hexagonal Close-Packed Crystals

He et al.

This PDF file includes:

Supplementary Figures 1-5

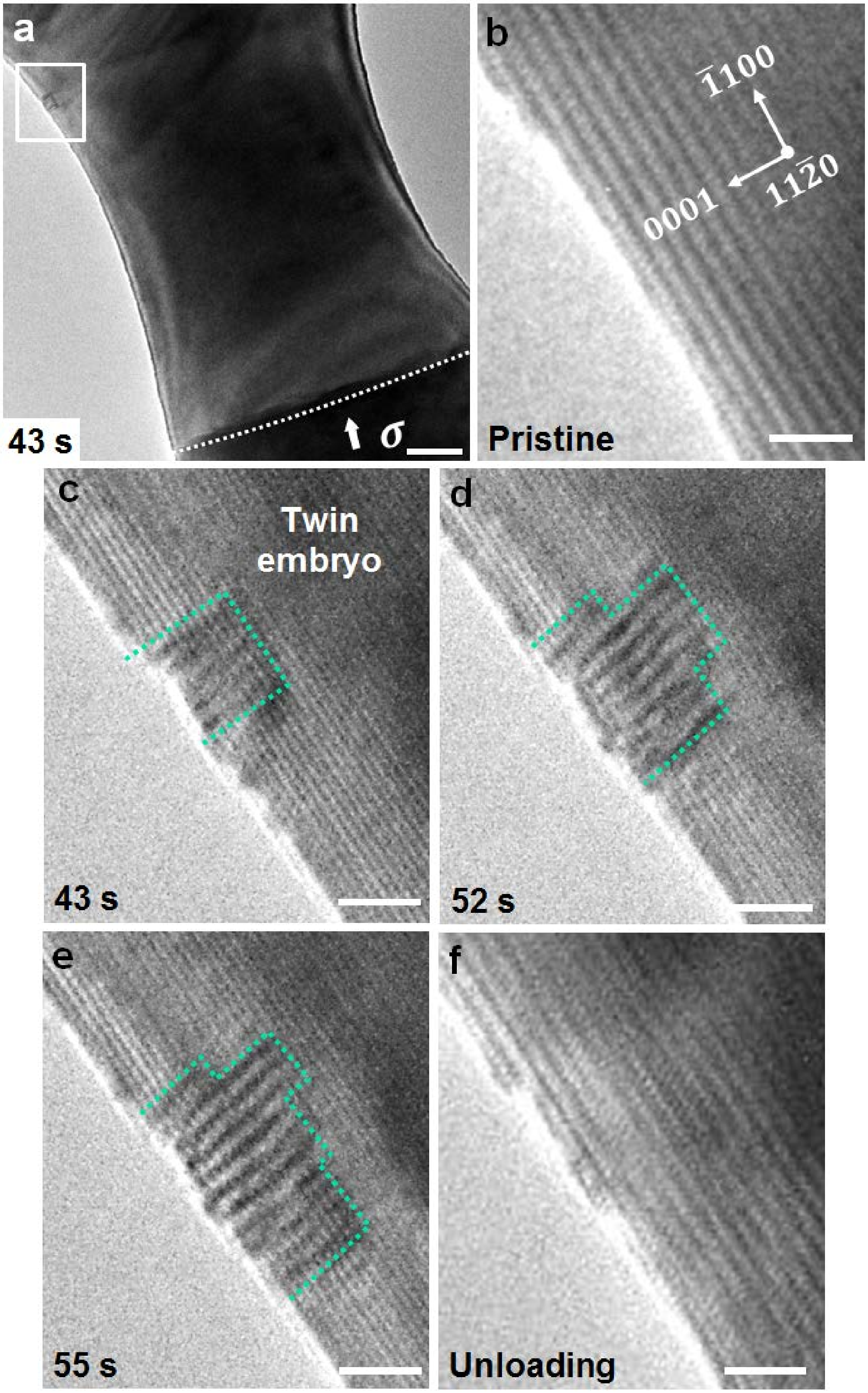
a
43 s
σ
b
$\bar{1}100$
0001
$11\bar{2}0$
Pristine
c
Twin
embryo
43 s
d
52 s
e
55 s
f
Unloading

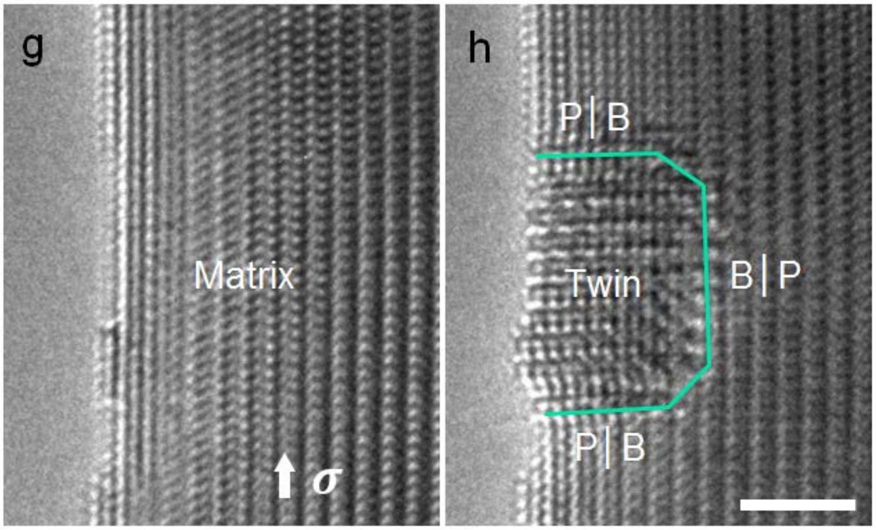

Supplementary Figure 1. Twinning nucleation on the free surfaces of rhenium nanocrystals under <1 -1 0 0>-oriented compression. (a) Low magnification TEM image of the rhenium nanocrystal under compression. Boxed area is the region of interest where twinning nucleation happens. (b-e) Sequential HRTEM snapshots showing the twinning nucleation at a local surface site, mediated by formation and lateral expansion of the twin Basal layers. White dotted lines indicate the P│B interfaces (twin boundary). Note that the surface site was defect free before twinning nucleation (b) and the twin embryo was unstable and instantly de-twined upon unloading (f). Instability of the twin embryo can be thermodynamically associated with the highly-strained state of the twin embryo (e.g. see Fig. 1d) and the high energies of P│B-type twin boundaries. (g-h) Sequential TEM snapshots showing another dataset on twinning nucleation from the side surface. Scale bars in (a, b-f, h), 5nm, 2nm, 2nm respectively.

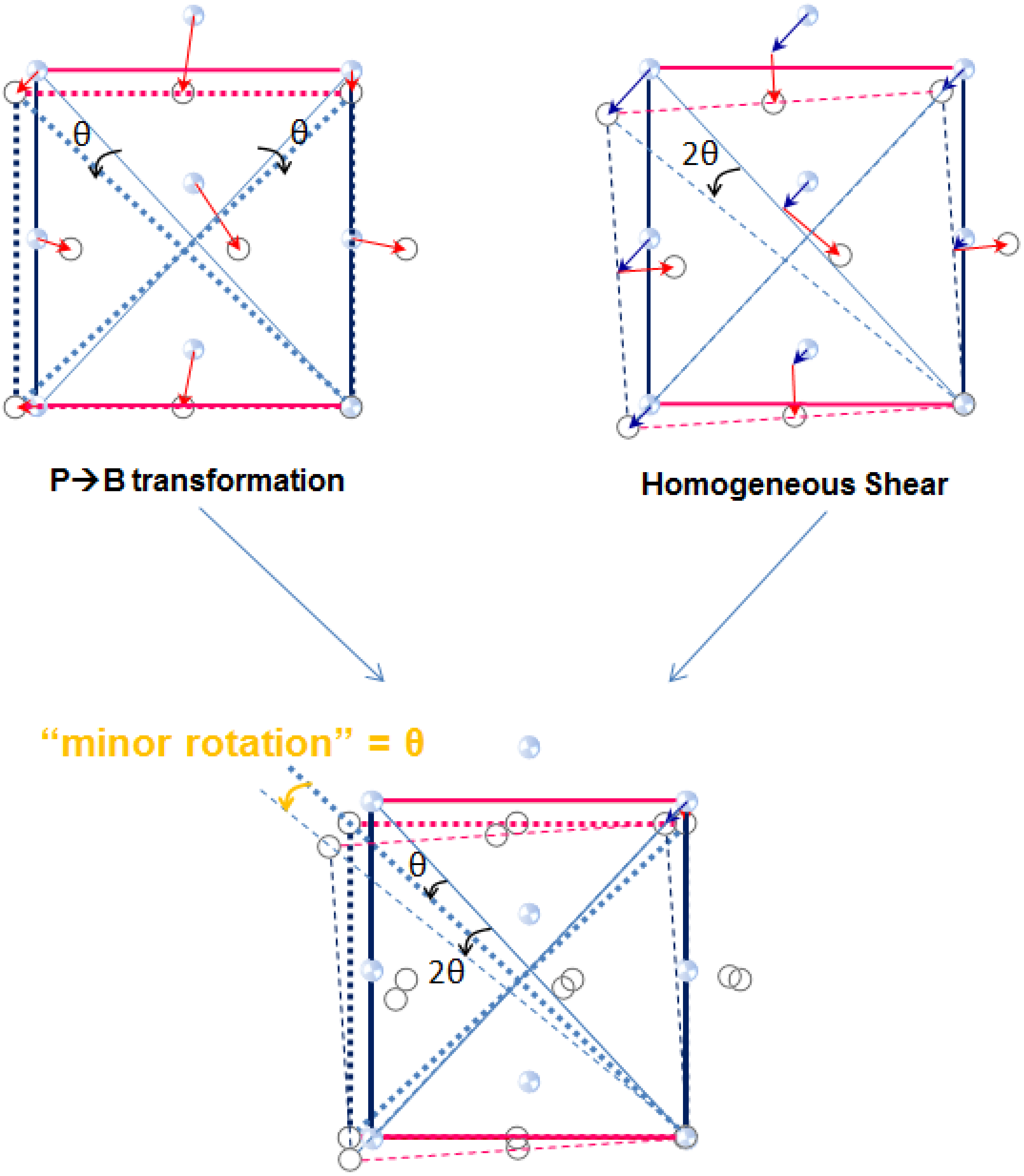

Supplementary Figure 2. Topological analysis of the magnitude of minor rotation between the twin lattices derived from the P→B transformation (denoted by thick dotted lines) and homogeneous-shear (denoted by thin dash lines). Clearly, the minor rotation should equal to θ which is exactly half of the rotation angle of K_2 plane in the homogeneous-shear model. Therefore, $\tan(\theta) = \frac{|\boldsymbol{b}_2|/2}{2 \cdot d_{\{10\bar{1}2\}}} = \frac{|\boldsymbol{s}|}{2}$, and the "minor rotation" (as shown in Fig. 3e-f) should be $\tan^{-1}(\boldsymbol{s}/2)$, which equals to 3.97^{o} (for Re) and is roughly the same as that measured in experiments (see Fig. 1d and Supplementary Fig. 3). Consequently, the ***P→B transformation-leaded "dual-step" twinning nucleation*** is

energetically more favorable when the twinning shear is small. As such, it is reasonable to apply the observed mechanism to Mg wherein {1 0 -1 2} twinning has a smaller shear than that in Re.

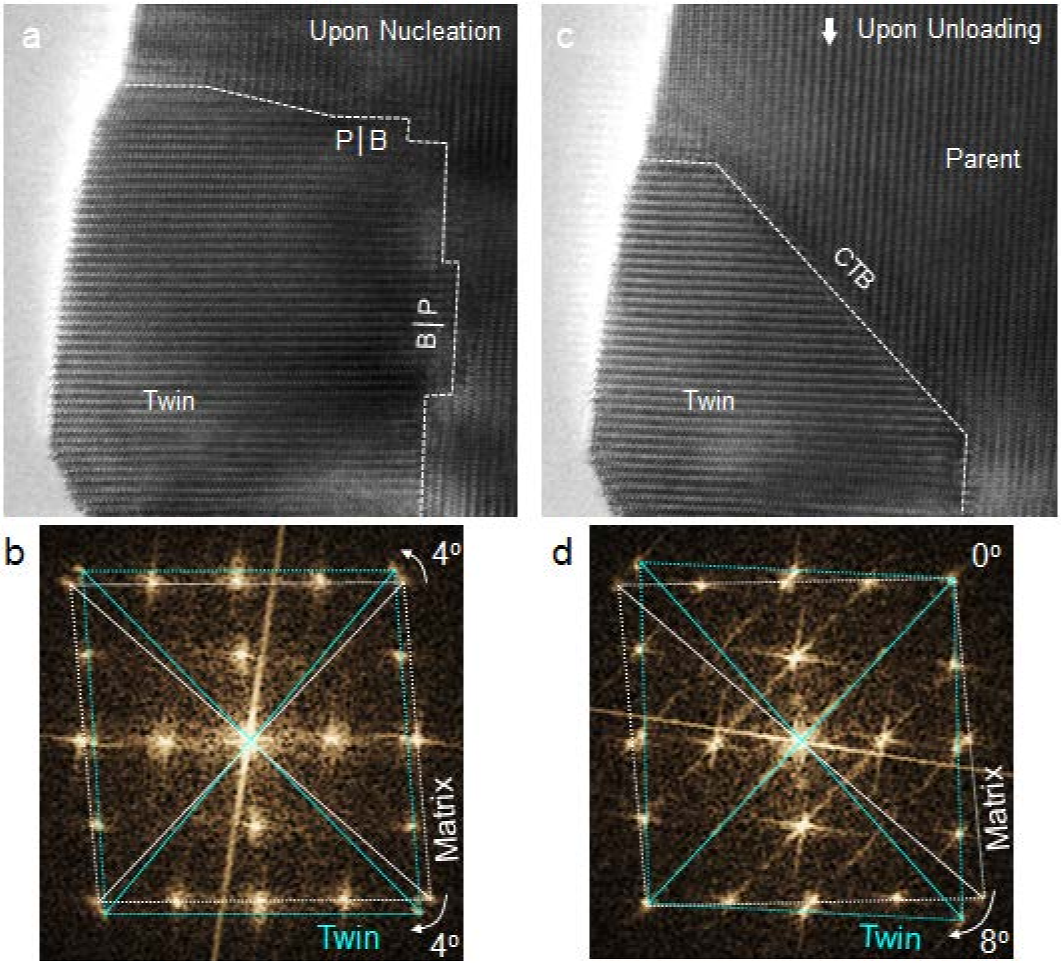

Supplementary Figure 3. Twin boundary transformation from Prismatic│Basal interfaces to coherent twin boundaries (CTB) and ensuing grain rotation upon unloading. White dash lines in (a, c) indicate the twin boundaries. White and cyan lines in (b, d) indicate the reciprocal lattices of the matrix and twin, respectively. Ensuing the transformation, the twin nucleus rotated 4° counter-clockwise and became an ideal twin.

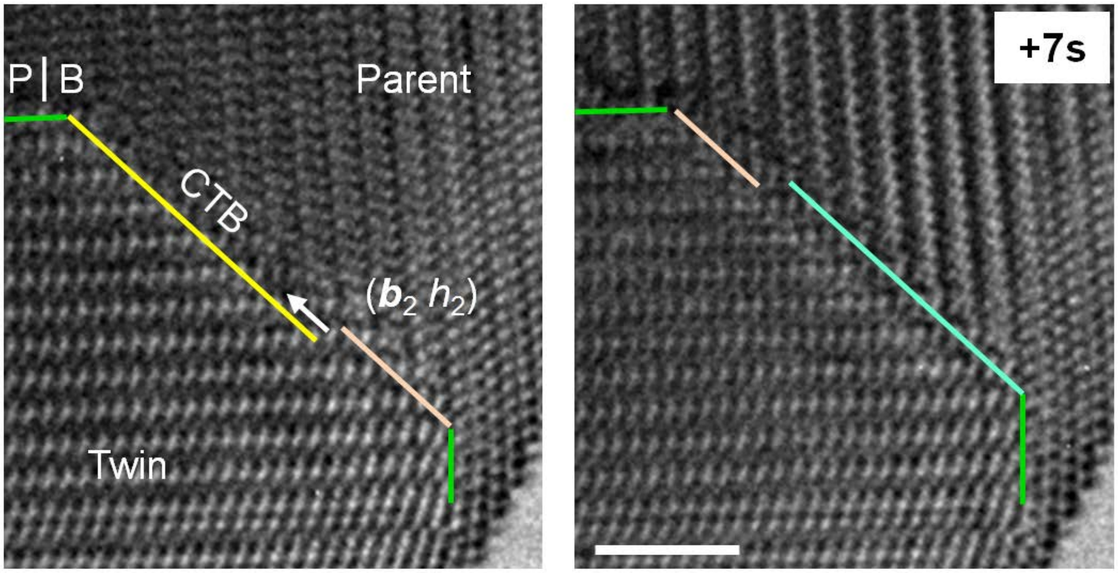

Supplementary Figure 4. Sequential TEM snapshots showing twinning dislocation-mediated coherent twin boundary migration during the twin (embryo) growth process. Scale bar, 2nm.

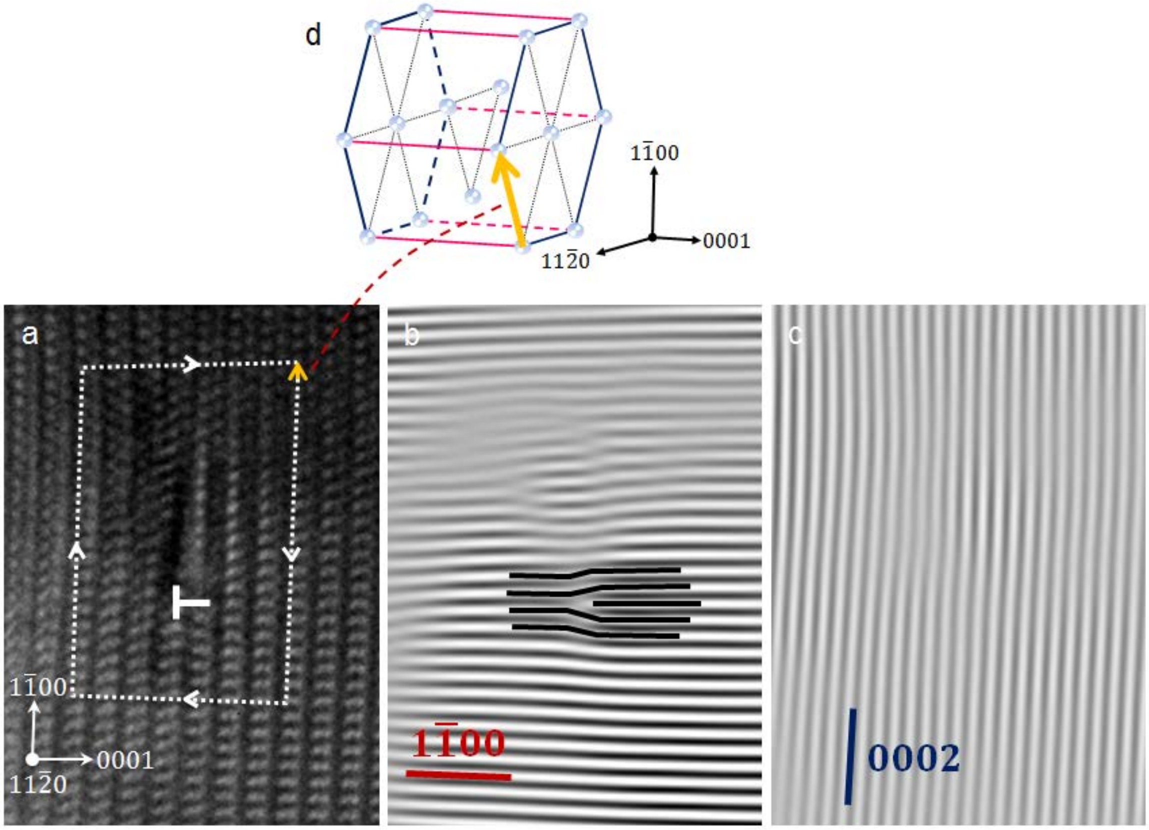

Supplementary Figure 5. Burgers vector analysis for the dislocation in Figure 5a of the main text. (a) HRTEM image reproduced from Fig. 5a. Dotted lines indicate the Burgers circuit. Yellow arrows indicate the projection of the Burgers vector on the (1 1 -2 0) view. (b, c) Inverse fast Fourier transformations of the HRTEM image showing only the (1 -1 0 0) and (0 0 0 2) planes, which further demonstrate that the dislocation contains no component along <***c***> axis. Black lines in panel (b) are to illustrate the extra half plane of the edge dislocation. (d) Model of the HCP unit-cell that illustrates the <***a***> Burgers vector in 3D.
