# Supplementary material for: Direct observation of dual-step twinning nucleation in hexagonal close-packed crystals
Source: Nat Commun. 2020 May 18;11:2483. doi: 10.1038/s41467-020-16351-0 (PMC7235251; doi:10.1038/s41467-020-16351-0)
Supplement: Supplementary file 2 — Description of Additional Supplementary Files [file 41467_2020_16351_MOESM2_ESM.docx]

Description for Additional Supplementary Files

**Supplementary Movie 1.** Twinning nucleation from the grain boundary of a rhenium bi-crystal under -oriented compression. The video was sped up 2 times.
